# Thrombelastography and biomarker profiles in acute coagulopathy of trauma: a prospective study

**DOI:** 10.1186/1757-7241-19-64

**Published:** 2011-10-26

**Authors:** Sisse R Ostrowski, Anne Marie Sørensen, Claus F Larsen, Pär I Johansson

**Affiliations:** 1 Section for Transfusion Medicine, Capital Region Blood Bank, Copenhagen University Hospital, Rigshospitalet, Blegdamsvej 9, DK-2100 Copenhagen, Denmark; 2 Department of Anesthesia, Copenhagen University Hospital, Rigshospitalet, Blegdamsvej 9, DK-2100 Copenhagen, Denmark; 3 The Trauma Centre, Centre of Head and Orthopedics, Copenhagen University Hospital, Rigshospitalet, Blegdamsvej 9, DK-2100 Copenhagen, Denmark

**Keywords:** Trauma, coagulopathy, trauma induced coagulopathy (TIC), Thrombelastography (TEG), platelets, fibrinogen, FXIII, sympathoadrenal activation, sCD40L

## Abstract

**Background:**

Severe injury induces an acute coagulopathy associated with increased mortality. This study compared the Thrombelastography (TEG) and biomarker profiles upon admission in trauma patients.

**Methods:**

Prospective observational study of 80 trauma patients admitted to a Level I Trauma Centre. Data on demography, biochemistry including standard coagulation tests, hematology, transfusions, Injury Severity Score (ISS) and TEG were recorded. Retrospective analysis of thawed plasma/serum for biomarkers reflecting tissue injury (histone-complexed DNA fragments), sympathoadrenal activation (adrenaline, noradrenaline), coagulation activation/inhibition and fibrinolysis (sCD40L, protein C, activated Protein C, tissue-type plasminogen activator, plasminogen activator inhibitor-1, D-dimer, prothrombinfragment 1+2, plasmin/α_2_-antiplasmin complex, thrombin/antithrombin complex, tissue factor pathway inhibitor, antithrombin, von willebrand factor, factor XIII). Comparison of patients stratified according to ISS/TEG maximum clot strength. Linear regression analysis of variables associated with clot strength.

**Results:**

Trauma patients had normal (86%), hypercoagulable (11%) or hypocoagulable (1%) TEG clot strength; one had primary hyperfibrinolysis. Hypercoagulable patients had higher age, fibrinogen and platelet count (all p < 0.05), none had increased activated partial thromboplastin time (APTT) or international normalized ratio (INR) and none required massive transfusion (> 10 red blood cells the initial 24 h). Patients with normal or hypercoagulable TEG clot strength had comparable biomarker profiles, but the few patients with hypocoagulable TEG clot strength and/or hyperfibrinolysis had very different biomarker profiles.

Increasing ISS was associated with higher levels of catecholamines, histone-complexed DNA fragments, sCD40L, activated protein C and D-dimer and reduced levels of non-activated protein C, antithrombin, fibrinogen and factor XIII (all p < 0.05). Fibrinogen and platelet count were associated independently with clot strength in patients with ISS ≤ 26 whereas only fibrinogen was associated independently with clot strength in patients with ISS > 26. In patients with ISS > 26, adrenaline and sCD40L were independently negatively associated with clot strength.

**Conclusions:**

Trauma patients displayed different coagulopathies by TEG and variables independently associated with clot strength changed with ISS. In the highest ISS group, adrenaline and sCD40L were independently negatively associated with clot strength indicating that these may contribute to acute coagulopathy.

## Background

Many severely injured patients develop an acute coagulopathy of trauma (ACT) already at the scene of the accident [1,2] and 25-35% are coagulopathic upon admission, a condition associated with a four-fold increase in mortality [3]. Previous studies have defined ACT as increases in plasma based coagulation tests (activated partial thromboplastin time (APTT), partial thrombin time (PTT), prothrombin time (PT), international normalized ratio (INR)) [4] but there is emerging evidence that the viscoelastic whole blood tests, Thrombelastography (TEG) and Rotation Thromboelastometry (ROTEM), can detect and discriminate between different types of traumatic coagulopathy [5] since this entity appears to change from normal to hypercoagulability, hypocoagulability and finally hyperfibrinolysis with increasing injury severity [1,6-11]. Immediate identification of the specific type of traumatic coagulopathy by TEG/ROTEM is of critical importance in order to goal-direct transfusion therapy and e.g. administer plasma, platelets, fibrinogen and/or antifibrinolytics to patients with evident hypocoagulability and/or hyperfibrinolysis [12,13]. We have used TEG to monitor hemostasis and guide transfusion therapy in massively bleeding patients since 2004 and this has significantly improved survival in these patients [14]. To our knowledge, no studies have directly compared outcomes in ratio-driven vs. TEG guided resuscitated bleeding trauma patients, and in addition to our previous finding of improved survival in bleeding patients resuscitated goal-directed according to TEG [14] we recently reported, in a meta-analysis of 16 studies of massively bleeding trauma patients, that the highest ratio of FFP and/or PLT to RBC was associated with a significantly reduced mortality (OR 0.49 (95% CI 0.43-0.57), p < 0.0001) as compared to the lowest ratio [15]. TEG/ROTEM were recently recommended internationally as gold standard point-of-care tests in bleeding trauma patients [16,17].

Though the exact pathophysiologic mechanism(s) of the ACT are unclear, retrospective analyses of circulating biomarkers have pointed to downstream effects of tissue injury, sympathoadrenal activation and hypovolemic/hemorrhagic shock as drivers [3,4,18] of an enhanced early protein C (PC) activation, hyperfibrinolysis [3,4,18] and endothelial damage [19], which may all contribute to the coagulopathy. Despite the recognized differences in presenting TEG/ROTEM profile in trauma patients [5], no studies have so far reported on circulating biomarker levels of tissue injury, sympathoadrenal activation and coagulopathy in trauma patients with different TEG profiles.

The primary aim of this study was to investigate biomarkers of sympathoadrenal activation, tissue injury, coagulation activation/inhibition and fibrinolysis in trauma patients stratified according to injury severity and TEG profile upon admission. A secondary aim was to identify biomarkers independently associated with TEG maximum clot strength as this parameter is the parameter most strongly associated with bleeding, transfusion requirements and outcome in massively bleeding patients [5]. We hypothesized that progressive coagulopathy by TEG (from normal to hypercoagulability, hypocoagulability and hyperfibrinolysis) would be accompanied by evidence of increased sympathoadrenal activation, tissue injury, PC activation and hyperfibrinolysis [20].

## Methods

### Study Design

Prospective observational cohort study of trauma patients admitted directly to a Level I Trauma Centre (TC) at a tertiary hospital (Rigshospitalet, Copenhagen, Denmark, covering 2.5 million inhabitants) between March 2010 and November 2010. The study is part of an ongoing larger multicentre study [21], Activation of Coagulation and Inflammation after Trauma 3 (ACIT3), approved by the Regional Ethics Committee (H-4-2009-139), the Danish Data Protection Agency and conducted in accordance with the 2nd Declaration of Helsinki. Written informed consent was obtained from the patients or next of kin. Here we report on preliminary findings related to a cohort of 80 patients recruited to the ACIT3 study.

### Patient selection

ACIT3 study inclusions: Adult trauma patients (≥ 18 years) who met criteria for full trauma team activation *and *had an arterial cannula inserted. The latter was chosen since only patients with expected severe injuries have an arterial cannula placed immediately upon TC admission. Exclusion criteria, according to the multicentre study protocol [21], were arrival in the TC > 2 hours after injury; > 2,000 ml of intravenous fluids administered before hospital arrival; transfer from another hospital or burns > 5% total body surface area. Patients were retrospectively excluded if they were taking anticoagulant/antiplatelet medications (except aspirin); had moderate or severe liver disease or had known bleeding diathesis.

The 80 included patients were selected from the first 100 patients recruited to the ACIT3 study with complete data. We intended to include 80 patients because we measured an extensive number of biomarkers by ELISA, with each ELISA kit providing analysis of 80 samples. We aimed at including the most severely injured and/or coagulopathic patients and selected the 80 patients according to: Outcome (mortality or ICU admission post trauma; yes), transfusion of RBC within 6 hours (yes), RTS (< 5.00, we had not access to ISS before later in the study period) or coagulopathy (APTT ≥ 35 sec, INR ≥ 1.2, Ly30 > 1%/Cl30 < 95%; yes). This yielded 70 severely injured/coagulopathic patients, and additionally 10 patients (age 48 years (IQR 43-52), 60% males) were selected blinded from the remaining 30 patients to match their age and gender (see Table 1 for details on demography, injury severity etc.). The 20 patients not included in this study, had, compared to the included patients, comparable age and gender (41 years (IQR 33-53), 40% males) and APTT (26 (IQR 23-27), NS) but had, as expected, lower ISS (4 (IQR 2-10), p < 0.001), mortality (0%, p = 0.037) and INR (1.1 (IQR 1.0-1.1), p = 0.007). Two of the 20 patients not included had a hypercoagulable TEG (MA > 69, 10%).

**Table 1:** Demography, injury severity, transfusion requirements, mortality, biochemistry and hemostasis, thrombelastography and biomarkers of coagulopathy in 80 trauma patients stratified according to injury severity score (ISS)

		ISS > 26	ISS 15-26	ISS < 15	p-value	
**Demography, injury severity, transfusion and mortality**						
N		23	26	30		
Age	yrs	45 (25-71)	57 (43-65)	42 (28-49)	**0.034**	^c^
Gender	m% (n)	74% (17)	62% (16)	67% (20)	NS	
Blunt trauma	% (n)	96% (22)	96% (25)	83% (25)	0.142	
ISS	score	34 (29-36)	22 (17-25)	9 (5-10)	NA	
sTBI	% (n)	17% (4)	46% (12)	29% (6)	0.091	
GCS (PH)	score	7 (3-13)	11 (6-15)	15 (12-15)	**0.002**	^a^
Crystalloids (PH)	ml	900 (250-1250)	275 (0-500)	250 (25-750)	**0.013**	^a, b^
RBC 0-24 h	units	2 (0-12)	1 (0-3)	0 (0-1)	**0.007**	^a^
FFP 0-24 h	units	2 (0-9)	0 (0-1)	0 (0-0)	**0.003**	^a^
PLT 0-24 h	units	0 (0-4)	0 (0-0)	0 (0-0)	**0.006**	^a^
MT (> 10 RBC in 24 h)	% (n)	35% (8)	4% (1)	3% (1)	**0.001**	^a, b^
Mortality	% (n)	39% (9)	15% (4)	3% (1)	**0.003**	^a^
**Shock, sympathoadrenal activation, cell damage, biochemistry and hemostasis**						
SBP (PH)	mmHg	150 (108-156)	136 (125-152)	130 (123-143)	NS	
pH		7.31 (7.25-7.33)	7.40 (7.307.40)	7.37 (7.33-7.41)	**0.001**	^a, b^
Lactate	mmol/l	1.7 (1.2-2.2)	2.5 (1.3-3.1)	1.5 (0.9-2.1)	**0.049**	^c^
SBE	mmol/l	-3.6 (-5.7- -1.9)	-2.7 (-4.6- -0.5)	-1.0 (-1.8-0.7)	**0.005**	^a^
Adrenaline	pg/ml	1,062 (328-1,549)	292 (126-1,077)	247 (86-354)	**0.002**	^a^
Noradrenaline	pg/ml	1,235 (434-1,511)	652 (210-1,288)	332 (217-1,036)	**0.040**	^a^
hcDNA	%	16.9 (5.6-27.0)	5.6 (2.4-10.3)	0.4 (0.0-9.3)	**<0.001**	^a^
Hemoglobin	mmol/l	8.0 (6.3-9.2)	8.6 (7.2-9.1)	8.4 (7.7-8.9)	NS	
Platelet count	10^9^/l	217 (165-252)	193 (173-260)	211 (191-240)	NS	
Fibrinogen	g/l	2.0 (1.4-2.2)	2.4 (2.1-2.9)	2.6 (2.3-2.9)	**0.002**	^a, b^
FXIII	microg/ml	25 (20-30)	30 (22-40)	32 (28-41)	**0.001**	^a^
vWF	%	190 (110-218)	200 (132-223)	204 (146-230)	NS	
APTT > 35 sec	%	22% (5)	4% (1)	0%	**0.009**	^a^
INR > 1.2	%	43% (10)	0%	0%	**<0.001**	^a, b^
**Thrombelastography**						
R	min	5.1 (4.8-5.9)	5.9 (5.1-6.7)	6.0 (5.3-6.4)	**0.046**	
Angle	degrees	65 (62-68)	67 (62-7)	66 (62-70)	NS	
MA	mm	63 (58-67)	64 (62-68)	63 (61-67)	NS	
Ly30	%	0.2 (0.0-0.7)	0.0 (0.0-0.2)	0.3 (0.0-1.0)	**0.040**	
**Platelet activation and thrombin generation**						
sCD40L	pg/ml	394 (281-551)	327 (251-406)	250 (195-305)	**0.006**	^a^
PF1.2	nmol/l	4.1 (1.1-13.0)	15.1 (4.1-45.3)	4.2 (1.6-9.5)	**0.026**	^b, c^
TAT	ng/ml	36 (32-41)	39 (33-44)	35 (29-40)	0.181	
**Natural anticoagulation**						
AT	10^3 ^U/l	0.89 (0.69-0.96)	0.9 (0.8-1.00)	0.98 (0.90-1.07)	**0.002**	^a^
PC	%	92 (75-116)	107 (96-123)	117 (100-129)	**0.031**	^a^
APC	ng/ml	10.4 (9.4-12.1)	10.5 (8.7-13.5)	8.0 (6.8-10.5)	**0.005**	^a, c^
TFPI	ng/ml	64 (48-86)	67 (52-80)	54 (41-74)	NS	
**Fibrinolysis**						
D-dimer	ng/ml	173 (172-176)	170 (144-175)	128 (36-148)	**<0.001**	^a, c^
tPA	ng/ml	7.3 (5.5-15.4)	7.8 (4.7-13.7)	5.2 (2.0-9.9)	0.063	
PAI-1	ng/ml	24 (11-37)	31 (14-68)	20 (13-27)	0.145	
PAP	ng/ml	316 (22-599)	475 (83-1152)	225 (54-393)	0.159	

Data are presented as medians (IQR) or n (%), with p-values shown for variables with p < 0.200, and in bold for p < 0.050. ISS groups were compared by Kruskal-Wallis and Bonferroni adjusted Wilcoxon Rank Sum post hoc tests, Wilcoxon Rank Sum tests and Chi-square/Fischer exact tests, as appropriate.

^a^ISS gr. 0 (ISS < 15) vs. 2 (ISS > 26) Bonferroni adjusted p < 0.05; ^b^ISS gr. 1 (ISS 15-26) vs. 2 (ISS > 26) Bonferroni adjusted p < 0.05; ^c^ISS gr. 0 (ISS < 15) vs. 1 (ISS 15-26) Bonferroni adjusted p < 0.05.

ISS, injury severity score; sTBI, severe Traumatic Brain Injury, Abbreviated Injury Score head > 3; PH, pre-hospital at the site of injury; GCS, Glascow Coma Score scale; RBC, red blood cells; FFP, fresh frozen plasma; PLT, platelet concentrates; MT, > 10 RBC the initial 24 hours; SBP, systolic blood pressure; APTT, activated partial thromboplastin time; INR, international normalized ratio. Biomarker abbreviations, see Materials and Methods section, ELISA.

Data on demography, clinical and biochemical parameters, investigations, management and 30-day mortality were recorded and ISS scores were obtained from the Trauma Audit & Research Network (TARN) database.

No patients received Tranexamic acid or catecholamines (Adrenaline or Noradrenaline) for hemodynamic stabilization prior to blood sampling.

### Blood sampling

Blood was sampled immediately upon arrival for standard arterial blood gas (ABG, Radiometer ABL 725/735, Copenhagen, Denmark), routine biochemistry and research analyses (citrate, heparin, EDTA plasma, serum). Routine biochemistry samples were analyzed in a DS/EN ISO 15189 standardized laboratory by a Sysmex XE-2100 (hemoblobin, platelets, leukocytes) and ACL TOP (APTT, INR, AT, fibrinogen). Samples for functional hemostatic assays were kept at room temperature (RT) until analyzed precisely 1 h after sampling. Plasma samples were ice-cooled immediately whereas serum samples were kept at RT for 1 h before centrifugation (one (serum) or two (plasma) times 1800 g at 5°C for 10 min) and storage at -80°C.

### Enzyme linked immunosorbent assay (ELISA) measurements

Soluble biomarkers of tissue injury, sympathoadrenal activation, coagulation activation/inhibition and fibrinolysis were measured in uniplicate by commercially available immunoassays according to the manufactures recommendations. In each patient, a total of 15 biomarkers were measured corresponding to a total of 15*80 = 1,280 measurements, with only 3 missing measurements. The biomarkers were analyzed in EDTA or citrate plasma as follows: EDTA plasma: adrenaline and noradrenaline (2-CAT ELISA, Labor Diagnostica Nord GmbH & Co. KG, Nordhorn, Germany; lower limit of detection (LLD) 11 pg/ml (adrenaline, normal reference < 100 pg/ml) and 44 pg/ml (noradrenaline, normal reference < 600 pg/ml), respectively. Histone-complexed DNA fragments (hcDNA, Cell Death Detection ELISA^PLUS^, Roche, Hvidovre, Denmark; LLD not stated, relative quantification); D-dimer (ADI; LLD 2-4 ng/ml) and sCD40L (R&D Systems Europe; LLD 4.2 pg/ml). Citrate plasma: protein C (PC, Helena Laboratories, Beaumont, TX, US; LLD 5% of reference plasma); activated protein C (APC, USCNLIFE; LLD 4.2 pg/ml); tissue-type plasminogen activator (tPA, ADI, detects sc-tPA, tc-tPA and tPA/PAI-1 complexes; LLD 1 ng/ml); plasminogen activator inhibitor-1 (PAI-1, Assaypro; LLD 0.2 ng/ml); prothrombinfragment 1 and 2 (PF1.2, USCNLIFE; LLD 0.043 nmol/l); plasmin/α_2_-antiplasmin complex (PAP, ADI; LLD not stated); thrombin/antithrombin complex (TAT, USCNLIFE; LLD 0.215 ng/ml); tissue factor pathway inhibitor (TFPI, ADI, detects intact TFPI, truncated TFPI, TF/FVIIa/TFPI complexes; LLD 0.18 ng/ml); von Willebrand Factor antigen (vWF, Helena Laboratories, LLD 5% of reference plasma); factor XIII (FXIII, Assaypro; LLD 50 pg/ml).

### Thrombelastography (TEG)

Whole blood clot formation was assessed in 3.2% citrated whole blood using a TEG^® ^5000 Hemostasis Analyzer System (Haemonetics Corp., MA, US), according to the manufacturers recommendations. All analyses were conducted within 2 hours from blood sampling at 37°C. The variables recorded were [normal range reported by Haemonetics Corp.]: reaction time (R [3-8 min], rate of initial fibrin formation), angle (α [55-78 degrees], clot growth kinetics, reflecting the thrombin burst), maximum amplitude (MA, clot strength [51-69 mm], reflecting maximum clot strength) and lysis after 30 min (Ly30 [0-8%], proportional reduction in the amplitude after MA, reflecting fibrinolysis) [5]. Patients were stratified according to TEG MA into the following groups: Normocoagulable (MA from 51-69 mm, n = 69), hypercoagulable (MA > 69 mm, n = 9), hypocoagulable (MA < 51, n = 1) and hypocoagulable hyperfibrinolysis (MA < 51 and Ly30 > 8%, n = 1). The day-to-day CV% of TEG MA is < 7% in our laboratory [22].

### Statistics

Statistical analysis was performed using SAS 9.1 (SAS Institute Inc., Cary, NC, US). Data from patients stratified according to ISS group (ISS > 26, ISS 15-26, ISS < 15) or maximum clot strength (TEG MA, normal vs. hypercoagulable) were compared by Kruskal-Wallis and Bonferroni adjusted Wilcoxon Rank Sum post hoc tests, Wilcoxon Rank Sum tests and Chi-square/Fischer exact tests, as appropriate. The contribution of platelets, fibrinogen, FXIII and biomarkers to the variation in maximum clot strength was investigated separately in each ISS group by univariate and multivariate linear regression analysis. Data are presented as medians with inter quartile ranges (IQR). P-values < 0.05 were considered significant.

## Results

### Study patients

The 80 patients presented with ISS in the entire range (ISS > 26 n = 23, 15-26 n = 26 and < 15 n = 30), with demography, injuries, transfusion requirements, mortality, biochemistry, thrombelastography and biomarkers as depicted in Table 1. Most patients (96%) were referred by mobile emergency care units (MECU) staffed with anesthetists (26% by helicopter) and blood samples were drawn a median of 68 min (IQR 48-88) after the injury. Increasing ISS was associated with higher mortality (18% overall mortality), lower Glascow Coma Score scale, increased volume of prehospital crystalloids and higher blood transfusion requirements, catecholamines, biomarkers of tissue injury and shock (pH, lactate, SBE) (Table 1).

Mortality causes were in brief: Of the 11 patients whom expired in the group with normal TEG, eight died within 24 h (50% from severe (s) TBI) and three died on days 7, 7 and 24 post-injury, two from sTBI sequels. The two patients who expired in the group with hypercoagulable TEG died days 7 and 8 post-injury, one from sTBI sequels. The patients with hyperfibrinolysis died within 24 h from severe non-TBI injuries.

### Injury severity and coagulopathy

ACT defined by APTT or INR above normal, were present in 15% of all patients (8% and 13% had increased APTT and INR, respectively) with increasing prevalence in the highest ISS group (Table 1). Furthermore, increasing ISS was associated with reduced fibrinogen and FXIII levels and also with reduced TEG R time and increased Ly30.

With regards to biomarkers of coagulation activation, increasing ISS was associated with increased sCD40L, a biomarker of platelet activation, and with significantly increased PF1.2 in moderately injured patients (ISS 15-26) as compared to both more severely and less injured patients. A similar tendency was observed for TAT (Table 1). Considering biomarkers of natural anticoagulation and fibrinolysis, AT and non-activated PC declined with increasing ISS whereas APC and D-dimer increased (Table 1).

### Maximum clot strength and biomarkers of coagulopathy

When stratifying patients according to normal (n = 69) vs. hypercoagulable (n = 9, 11%) clot strength (TEG MA), patients with hypercoagulable clot strength were older, had a higher platelet count and fibrinogen level and also tended to have a higher FXIII level (Table 2). No patients in the hypercoagulable group had increased APTT or INR or required massive transfusion, and pre-hospital administration of fluids was comparable in the patients with normal or hypercoagulable clot strength (Table 2). Furthermore, hypercoagulable patients had faster clot growth kinetics (TEG angle, reflecting the thrombin burst) and a tendency towards reduced fibrinolysis (TEG Ly30) (Table 2).

**Table 2:** Demography, injury severity, transfusion requirements, mortality, biochemistry and hemostasis, thrombelastography and biomarkers of coagulopathy in 80 trauma patients stratified according to TEG profile (normal, hypercoagulability, hypocoagulability, hyperfibrinolysis)

		Normal	Hyper- coagulable	Hypo- coagulable	Hyper- fibrinolysis	p-value^a^
		(51-69 mm)	(> 69 mm)	(< 51 mm)	(< 51 mm/> 8%)	
**Demography, injury severity, transfusion and mortality**						
N		69	9	1	1	
Age	yrs	44 (32-58)	72 (59-80)	63	80	**0.006**
Gender	m% (n)	68% (47)	56% (5)	100%	100%	NS
Blunt trauma	% (n)	90% (62)	100%	100%	100%	NS
ISS	score	18 (10-29)	17 (16-20)	25	50	NS
sTBI	% (n)	30% (18)	33% (3)	100%	0%	NS
GCS (PH)	score	13 (6-15)	12 (9-14)	7	6	NS
Crystalloids (PH)	ml	300 (0-1000)	500 (200-500)	0	500	NS
RBC 0-24 h	units	0 (0-4)	0 (0-1)	0	45	NS
FFP 0-24 h	units	0 (0-3)	0 (0-0)	0	36	NS
PLT 0-24 h	units	0 (0-1)	0 (0-0)	0	15	NS
MT (> 10 RBC in 24 h)	% (n)	14% (10)	0%	0%	100%	0.159
Mortality	% (n)	16% (11)	22% (2)	0%	100%	NS
**Shock, sympathoadrenal activation, cell damage, biochemistry and hemostasis**						
SBP (PH)	mmHg	135 (122-149)	129 (100-153)	170	95	NS
pH		7.34 (7.29-7.38)	7.37 (7.31-7.41)	7.38	7.06	NS
Lactate	mmol/l	1.9 (1.4-2.9)	2.3 (1.4-2.8)	1.3	8.4	NS
SBE	mmol/l	-2.1 (-4.1--0.2)	0.3 (-2.0-0.7)	-1.9	-10.7	0.127
Adrenaline	pg/ml	295 (112-976)	1,163 (191-1,471)	298	5,427	0.169
Noradrenaline	pg/ml	638 (276-1,307)	1,146 (120-2,540)	731	549	NS
HcDNA	%	4.7 (0.1-13.5)	10.0 (7.2-16.4)	9.8	17.1	0.126
Hemoblobin	mmol/l	8.4 (7.6-9.1)	7.5 (6.5-8.8)	8.8	6.4	NS
Platelet count	10^9^/l	206 (173-251)	275 (185-306)	117	172	**0.045**
Fibrinogen	g/l	2.3 (2.0-2.7)	3.1 (3.0-3.4)	1.1	1.1	**< 0.001**
FXIII	microg/ml	29 (24-36)	37 (29-43)	21	18	0.096
vWF	%	200 (127-225)	190 (151-222)	124	203	NS
APTT > 35 sec	% (n)	7% (5)	0%	0%	100%	NS
INR > 1.2	% (n)	13% (9)	0%	0%	100%	0.134
**Thrombelastography**						
R	min	5.6 (4.9-6.4)	5.4 (5.1-5.7)	6.7	6.1	NS
Angle	degrees	65 (62-69)	70 (67-73)	41	54	**0.027**
MA	mm	63 (61-66)	71 (69-73)	49	33	NA
Ly30	%	0.2 (0.0-0.6)	0.0 (0.0-0.0)	0	78	0.076
**Platelet activation and thrombin generation**						
sCD40L	pg/ml	292 (223-417)	332 (263-406)	399	1,431	NS
PF1.2	nmol/l	6.6 (2.7-18.0)	4.5 (1.0-22.4)	1.0	0.4	NS
TAT	ng/ml	37 (31-43)	36 (31-44)	29	38	NS
**Natural anticoagulation**						
AT	10^3 ^U/l	0.92 (0.82-1.02)	0.95 (0.88-0.98)	0.98	0.61	NS
PC	%	108 (91-124)	108 (100-135)	138	71	NS
APC	ng/ml	9.8 (7.6-12.1)	9.3 (9.1-11.1)	13.7	10.3	NS
TFPI	ng/ml	58 (44-78)	78 (57-84)	126	68	0.075
**Fibrinolysis**						
D-dimer	ng/ml	166 (122-173)	170 (141-174)	179	173	NS
tPA	ng/ml	6.9 (3.6-12.0)	12.1 (5.8-14.8)	2.6	25.4	0.123
PAI-1	ng/ml	22 (12-38)	33 (24-68)	8	24	NS
PAP	ng/ml	283 (45-619)	510 (331-1154)	0	316	0.126

Data are presented as medians (IQR) or n (%), with p-values shown for variables with p < 0.200, and in bold for p < 0.050.

^a^Only patients with normal or hypercoagulable TEG MA were compared (Wilcoxon Rank Sum tests or Chi-square/Fischer exact tests, as appropriate) but data are presented on the two patients that displayed hypocoagulability (MA < 51 mm) and hypocoagulable hyperfibrinolysis (MA < 51 mm and Ly30 > 8%).

ISS, injury severity score; sTBI, severe Traumatic Brain Injury, Abbreviated Injury Score head > 3; PH, pre-hospital at the site of injury; GCS, Glascow Coma Score scale; RBC, red blood cells; FFP, fresh frozen plasma; PLT, platelet concentrates; MT, > 10 RBC the initial 24 hours; SBP, systolic blood pressure; APTT, activated partial thromboplastin time; INR, international normalized ratio. Biomarker abbreviations, see Materials and Methods section, ELISA.

Two patients presented with hypocoagulability and primary hyperfibrinolysis, respectively, and these are displayed separately in Table 2 for comparison, though no attempt was done to statistically compare these patients with the normal or hypercoagulable groups. It is notable that fibrinogen, FXIII and thrombin generation (PF1.2) was profoundly reduced in these two patients and that adrenaline, hcDNA, sCD40L and tPA was markedly increased in the patient with primary hyperfibrinolysis (Table 2).

### Injury severity and predictors of maximum clot strength

Given that platelets, fibrinogen and FXIII contribute significantly to TEG clot strength [23-25], we investigated the association between these variables and maximum clot strength in patients stratified according to ISS and also investigated the influence of the measured biomarkers on the independent association between fibrinogen, platelet count, FXIII and clot strength (Table 3).

**Table 3:** Univariate and multivariate contribution of fibrinogen, platelets, FXIII and sCD40L to the variation in TEG maximum amplitude (MA) in trauma patients stratified according to ISS (gr. 2 ISS > 26, gr. 1 ISS 15-26, gr. 0 ISS < 15)

		Univariate		Multivariate (model 1)		Multivariate (model 2)	
	Unit	β (SE)	p	β (SE)	p	β (SE)	p
**ISS > 26 (n = 23)**				**R^2 ^= 0.47**		**R^2 ^= 0.76**	
Fibrinogen	1 g/l	6.59 (1.85)	**0.002**	5.61 (1.86)	**0.007**	4.65 (1.31)	**0.002**
Platelets	10 *10^9^/l	0.48 (0.28)	0.094	0.29 (0.25)	0.438	0.42 (0.18)	**0.032**
FXIII	1%	0.43 (0.20)	**0.043**	0.24 (0.19)	0.212	0.17 (0.13)	0.210
sCD40L	100 pg/ml	-1.62 (0.53)	**0.006**	-	-	-1.62 (0.35)	**< 0.001**
**ISS 15-26 (n = 26)**				**R^2 ^= 0.75**		**R^2 ^= 0.76**	
Fibrinogen	1 g/l	6.84 (1.08)	**< 0.001**	6.33 (0.96)	**< 0.001**	5.84 (1.04)	**< 0.001**
Platelets	10 *10^9^/l	0.41 (0.20)	**0.047**	0.37 (0.13)	**0.011**	0.44 (0.14)	**0.006**
FXIII	1%	0.13 (0.11)	0.245	0.01 (0.07)	0.847	0.03 (0.07)	0.712
sCD40L	100 pg/ml	-0.95 (0.89)	0.300	-	-	-0.73 (0.61)	0.250
**ISS < 15 (n = 30)**				**R^2 ^= 0.41**		**R^2 ^= 0.41**	
Fibrinogen	1 g/l	3.55 (1.12)	**0.004**	3.26 (1.05)	**0.005**	3.29 (1.07)	**0.005**
Platelets	10 *10^9^/l	0.34 (0.13)	**0.017**	0.30 (1.05)	**0.019**	0.28 (0.13)	**0.038**
FXIII	1%	0.00 (0.04)	0.986	0.00 (0.03)	0.995	-0.00 (0.03)	0.984
sCD40L	100 pg/ml	0.38 (0.44)	0.403	-	-	0.15 (0.38)	0.691

Univariate and multivariate regression analysis showing regression coefficients (β) with standard errors (SE) and p-values, with p-values in bold for variables with p < 0.05. The β corresponds to the predicted change in TEG MA (mm) associated with one unit increase in fibrinogen, platelet count, FXIII and/or sCD40L.

By univariate linear regression analysis, fibrinogen was associated with clot strength in all ISS groups whereas FXIII was only associated with clot strength in the highest ISS group and platelets were only associated with clot strength in the lower ISS groups. When including fibrinogen, platelets and FXIII in a multivariate model (Model 1), fibrinogen was the only variable independently associated with clot strength in the highest ISS groups whereas both fibrinogen and platelets were independently associated with clot strength in the two lower ISS groups (Table 3).

When confronting the multivariate model (fibrinogen, platelets, FXIII) with the measured biomarkers, sCD40L was independently negatively associated with clot strength only in the highest ISS group and importantly, inclusion of sCD40L made platelets independently positively associated with clot strength also in these patients (Model 2, Table 3). Adrenaline was also independently negatively associated with clot strength only in the highest ISS group (β -2.20 (SE 0.81), p = 0.014; change in mm MA per 1 ng/ml increase in adrenaline) whereas APC was independently negatively associated with clot strength only in the lowest ISS group (β -0.53 SE), p = 0.009; change in mm MA per 1 ng/ml higher APC). None of the other measured biomarkers were independently associated with clot strength in any ISS groups.

## Discussion

The present study confirms that trauma patients present a spectrum of different coagulopathies that can be identified by TEG and demonstrates that hypercoagulable trauma patients are older and have higher fibrinogen level and platelet count. Furthermore, increasing injury severity was associated with increased shock, sympathoadrenal activation, tissue injury, platelet activation, protein C activation (higher activated PC, lower non-activated PC), hyperfibrinolysis and reduced fibrinogen and FXIII levels. Finally, in the most severely injured patients (highest ISS group), adrenaline and sCD40L were independently negatively associated with maximum clot strength and platelet count alone was only associated with clot strength after adjusting for platelet activation level (sCD40L).

Trauma is a leading cause of death and disability worldwide and hemorrhage is responsible for the majority of potentially preventable deaths. Death due to exsanguination occurs early (50% within 2 hours) after the injury [26] emphasizing that immediate diagnosis of existing coagulopathies by TEG/ROTEM is of critical importance to enable goal-directed therapy early in the resuscitation phase [12,14].

In accordance with previous studies [1,6-11] TEG identified a spectrum of different coagulopathies in trauma patients, with hypercoagulability being the most frequent [6] and in this study associated with high age and high fibrinogen level and platelet count. In contrast to some previous studies [6,11], but consistent with others [10], no difference in injury severity between patients with normal and hypercoagulable TEG was found. Though not statistically significant, it is notable that no hypercoagulable patients were massively transfused or had ACT according to APTT/INR, a finding in accordance with previous studies [6]. Furthermore, it is notable that the hypercoagulable and normal patients had comparable mortality despite a considerably higher age in the hypercoagulable group. It is tempting to speculate that a hypercoagulable response to moderate (survivable) trauma may be optimal from an evolutionary perspective by promoting hemostasis. Given this, the hypocoagulability and/or hyperfibrinolysis that may accompany severe (unsurvivable) injury may reflect a less well adapted exaggerated response to excessive systemic endothelial activation and damage along with extremely low flow and hypoperfusion [20].

The low prevalence of hyperfibrinolysis is in accordance with previous findings in trauma patients [1,7-9], but we found a lower than expected prevalence of hypocoagulability [1,6]. The latter may be due to the relatively low number of severely injured and/or shocked patients in the present study, which may also explain the low prevalence of patients with ACT according to APTT and INR. Though the 15% prevalence of ACT in the present study is within the range previously reported in other trauma studies (from 10-34%) [27], this relatively low proportion of patients with ACT should be taken into account when interpreting the results from the present study.

Though we lacked statistical power to compare patients with hypocoagulability or hyperfibrinolysis to patients with a normal TEG, the high level of sympathoadrenal activation, tissue injury, platelet activation, PC activation and tPA release in the patient with primary hyperfibrinolysis concurs with the biomarker profile expected to yield hyperfibrinolysis [3,4,20]. However, this finding reported here needs to be confirmed in a larger study powered to investigate biomarkers in patients with hypocoagulable or hyperfibrinolytic TEG profiles.

Increasing injury severity was associated with lower fibrinogen levels and importantly also with lower FXIII levels and higher prevalence of patients with ACT according to APTT or INR. The association between injury severity and fibrinogen, APTT and INR is well established [3,4] whereas the negative association between ISS and FXIII has not been described previously, despite an established association between low FXIII levels and increased bleeding following e.g., cardiac surgery and neurosurgery [28]. In accordance with previous studies, higher ISS was also associated with evidence of increased sympathoadrenal activation, tissue injury [18,19], PC activation and hyperfibrinolysis [3,4,18,19]. In contrast to previous studies of ACT reporting solely on decreases in non-activated PC as indirect evidence of PC activation [3,4], the present study is to our knowledge the first to directly demonstrate enhanced PC activation with increasing injury severity, evidenced by increased activated PC in the highest ISS groups, occurring along with a previously described decline in non-activated PC in these patients.

Though not statistically significant, the TEG profile changed towards reduced R time and increased fibrinolysis with increasing injury severity, a finding also in accordance with previous findings [6-8].

Maximum clot strength is a strong predictor of bleeding and transfusion requirements in trauma patients [6,10,11,29], explaining why we investigated variables independently associated with this. In the two lower ISS groups, the fibrinogen level and platelet count were both independently associated with clot strength, in accordance with previous findings [6,7,23,29]. Importantly, we found that the variables independently associated with clot strength changed with injury severity so that platelet count only remained independently associated with clot strength in the highest ISS group after adjusting for the platelet activation level (sCD40L). Based on this finding it could be speculated that trauma induced platelet activation (and ensuing release of sCD40L) may result in downstream platelet exhaustion or hypo responsiveness so the platelets left could not adequately support clot formation. Alternatively, the finding may simply reflect that adequately activated platelets were consumed in vivo upon clot formation leaving in the circulation (and collected upon blood sampling) platelets with lower hemostatic potential. The finding emphasizes that changes observed in the blood may optimally be interpreted from a systems biology perspective taking into account the condition of the vascular endothelium (activated, damaged, leaky etc.) surrounding the circulating blood and hence critically influencing the composition of both the circulating and sampled blood [20]. Whichever explanation, the notion that severe trauma may result in platelet exhaustion is in accordance with previous thoughts [30] and in accordance with a recent study of platelet function in trauma patients reporting on low platelet reactivity assessed by both Multiplate and by the platelet component of viscoelastic tests (ROTEM) in patients with highest ISS and non-survivors [31]. Finally, it should be noted that the fibrinogen values included in the statistical model in the present study may not adequately reflect fibrin polymerization (and hence functional contribution to clot strength) since optical measurements of circulating fibrinogen levels not always simply reflect fibrin polymerization.

Importantly, we also found that the adrenaline level was negatively independently associated with clot strength only in the highest ISS group indicating that excessive sympathoadrenal activation may negatively influence hemostasis. We recently proposed [20] and demonstrated [18] that progressive increases in adrenaline levels in trauma patients promote a switch from hypercoagulability towards hypocoagulability and hyperfibrinolysis due to the influence of adrenaline on the vascular endothelium [19,20]. The finding here supports this notion and emphasizes that the sympathoadrenal activation following trauma may contribute directly to the early coagulopathy observed in trauma patients.

Whether the different contribution of fibrinogen and platelets to TEG clot strength in patients with high vs. low injury severity reflects that these patients would benefit from different resuscitation strategies with e.g., FFP/fibrinogen/cryoprecipitate pool vs. platelets cannot be answered from the present explorative study but should be investigated in a randomized clinical trial powered to answer this question.

The results presented here are subject to the limitations inherent to observational studies and, thereby, do not allow independent evaluation of the cause-and-effect relationship suggested. Furthermore, the low number of subjects, and especially the low number of severely injured patients and patients with hypocoagulable or hyperfibrinolytic TEG profiles, included in the present study increases the risk of introducing a type II error, emphasizing that the reported findings should be confirmed in a larger cohort of patients. Finally, exclusion of patients based on pre-hospital fluid administration (> 2,000 ml) could theoretically have introduced a bias by excluding the most severely injured and bleeding patients, which should be taken into account. However, in 2004 our Trauma Centre introduced Hemostatic Control Resuscitation and abandoned colloids along with a general consensus of restrictive pre-hospital crystalloid fluid resuscitation so extremely few patients admitted to our Trauma Centre in the study period and today were resuscitated with > 2,000 ml fluids pre-hospital and no patients received colloids.

## Conclusions

This study demonstrates that trauma patients present with a range of coagulopathies when evaluated by TEG and that patients with hypercoagulable TEG are older and have higher fibrinogen level and platelet count. Increasing ISS was associated with more pronounced coagulopathy and importantly, also with increased activated protein C and reduced FXIII levels. The variables independently associated with clot strength changed with injury severity and in the most severely injured patients, adrenaline and sCD40L were independently negatively associated with maximum clot strength and platelet count was only associated with clot strength after adjusting for platelet activation level. The latter finding indicates that excessive sympathoadrenal activation and platelet activation in the most severely injured patients may contribute to the acute coagulopathy of trauma.

## Abbreviations

α: TEG angle; ACIT: activation of coagulation and inflammation after trauma; ACT: acute coagulopathy of trauma; APC: activated protein C; APTT: activated partial thromboplastin time; ELISA: enzyme linked immunosorbent assay; FXIII: factor XIII; hcDNA: histone-complexed DNA fragments; ICU: intensive care unit; INR: international normalized ratio; IQR: inter quartile range; Ly30: TEG lysis after 30 min; MA: TEG maximum amplitude; PAI-1: plasminogen activator inhibitor-1; PAP-complex: plasmin/α_2_-antiplasmin complex; PC: protein C; PF1.2: prothrombinfragment 1 and 2; R: TEG reaction time; ROTEM: rotation thromboelastometry; TARN: trauma audit & research network; TAT-complex: thrombin/antithrombin complex; TC: trauma centre; TEG: thrombelastography; TFPI: tissue factor pathway inhibitor; tPA: tissue-type plasminogen activator; vWF: von Willebrand Factor antigen.

## Competing interests

The authors declare that they have no competing interests.

## Authors' contributions

SRO contributed to the design of the study, analysis and interpretation of data, figure drafting and drafting/writing/revising of the manuscript. AMS and CFL contributed to the design of the study and revised the manuscript critically. PIJ contributed to the conception and design of the study, interpretation of data and drafting/writing/revising of the manuscript. All authors read and approved the final manuscript.
